# Photo-activated proflavine degrades protein and impairs enzyme activity: Involvement of hydroxyl radicals

**DOI:** 10.1016/j.toxrep.2021.12.009

**Published:** 2021-12-21

**Authors:** Mansour K. Ghatasheh, Abdul Malik, Mohammad Shamsul Ola, Abdullah S. Alhomida

**Affiliations:** a Department of Biochemistry, College of Science, King Saud University, P.O. Box 2455, Riyadh, 11451, Saudi Arabia; b Department of Pharmaceutics, College of Pharmacy, King Saud University, Riyadh, Saudi Arabia

**Keywords:** Proflavine, Reactive oxygen species, BSA, Trypsin, Hydroxyl radical

## Abstract

Proflavine is a well-known antiseptic and bacteriostatic drug, however, it has the potential to be hazardous and mutagenic. Proflavine enters cells and intercalates between DNA base pairs, resulting in mutation and replication inhibition. Previously several investigators demonstrated that photo-activated proflavine generated double-stranded DNA breakage and protein structural alterations. The present study investigated the role of hydroxyl radical (·OH) due to activation of proflavine in the breakdown of protein and enzyme by photo-activated proflavine. The results show that the formation of hydroxyl radicals increased as the photo-illumination period increased, as did the concentrations of proflavine and Cu (II). As demonstrated by SDS-PAGE, the excess of free radicals due to proflavine resulted in oxidative modifications and degradation of BSA protein and trypsin enzyme. Additionally, with an increase in Cu (II) concentration, photo-illuminated proflavine induced a considerable loss of enzyme activity and also accelerated the degradation of the enzyme. Bathocuproine, a particular Cu (I)-sequestering agent, prevented protein degradation and enzyme inactivation. Hydroxyl radical scavengers inhibited the protein-damaging process, indicating that hydroxyl radicals play a substantial role in protein damage. The tryptophan moiety was quenched by proflavine, demonstrating that it binds to proteins and enzymes, changing their structure and activity. As a result, this study helps to better understand proflavine's deleterious influence on protein and enzyme degradation by oxygen-free radicals.

## Highlights

• Generation of hydroxyl radical (·OH) increased by proflavine upon illumination with fluorescent light.
• Proflavine resulted in oxidative modifications and degradation of protein and enzyme structure.
• The addition of Cu (II) augmented photo-illuminated proflavine to generate hydroxyl radicals.
• Proflavine-induced hydroxyl radicals have a deleterious influence on protein and enzyme activity.

## Graphical abstract

**Figure:**
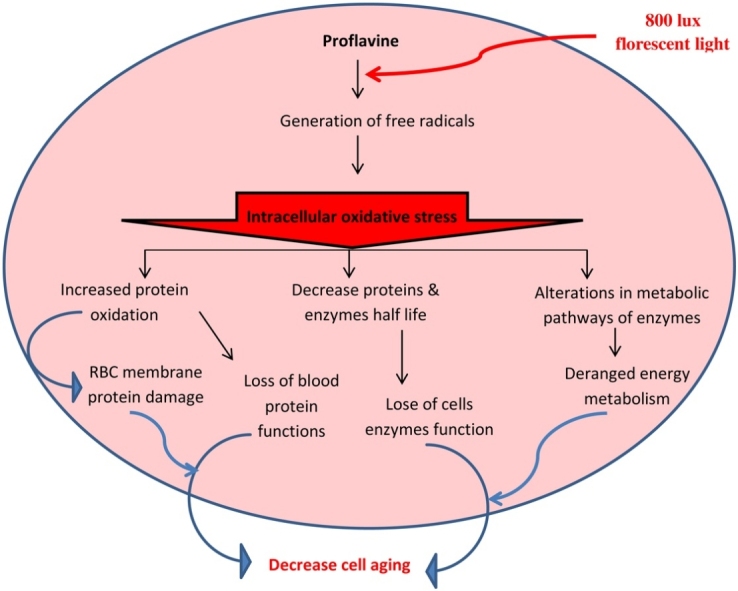

## 1 Introduction

Proflavine, an acridine dye, is commonly used as an antiseptic in wound treatments as well as bacterium disinfection. It was also discovered to be too toxic to be used as a systemic anti-bacterial [1,2]. Numerous publications showed frameshift mutations, when viruses, bacteria, cultured cells, and bacteriophages were treated with proflavine [[1], [2], [3]]. Furthermore, multiple investigations have revealed proflavine's mutagenic nature, as well as its significant hazard potential [2,4,5]. Proflavine can enter the epidermal and dermal layers of cells and concentrate in their nucleus [6,7]. This dye has also been discovered to impede cancer cell multiplication by intercalating between DNA base pairs [8,9]. Because of its capacity to interact with DNA double helix, it has the potential to be developed as an anticancer medication [[10], [11], [12]]. In vitro cytotoxicity tests on breast and ovarian cancer cell lines revealed that combining the medication with metal ions can result in a more effective antineoplastic agent [13]. When exposed to visible light, proflavine produces numerous types of reactive oxygen species, but only when a macromolecule like DNA is involved in the reaction [14]. Proflavine can generate double-stranded breaks in DNA in the presence of light, as we and others have previously discovered through free radical generation [4,6]. In addition, proflavine produced superoxide ion (O_2_^•^^−^) and caused structural changes in proteins, which was amplified when the divalent metal ion Cu (II) was added [15]. Copper ions are important trace element that is found throughout the body. The liver and kidney have a relatively high concentration of Cu (II), whereas typical serum contains up to 8 μM loosely bound copper that can be used in redox reactions especially in catalyzing the reactive oxygen species (ROS) formation [16,17].

Reactive oxygen species (ROS) accumulation can lead to oxidative stress, which has been linked to the development of a variety of chronic diseases and biological processes such as apoptosis, necrosis, autophagy, and inflammation [[18], [19], [20], [21], [22], [23]]. Superoxide anion production from proflavine may cause calf thymus and supercoiled plasmid DNA breakage, as well as protein degradation and red blood cell hemolysis [24,25]. Proflavine can produce (O_2_^•^^−^) through a direct interaction between an electron expelled by excited proflavine and molecular oxygen or through the decomposition of peroxide radical (^•^OOH). In the presence of H_2_O and O_2_, photoexcited proflavine can then give birth to ^3^O_2_ and ^1^O_2_ via direct energy transfer, as we previously stated can accept an electron from a molecule of oxygen and produce a peroxide radical. In the presence of H_2_O, this peroxide radical can produce hydroxyl radical (^•^OH) or ^•^OOH in the presence of copper. These free radicals may assault the target molecules and cause additional damage. DNA and protein damage, oxidation of polyunsaturated fatty acids, oxidation of amino acids, and deactivation of particular enzymes are all harmful effects of ROS, resulting in metabolic and cellular problems [26]. Proteins are the primary targets of ROS, which can play a regulating role in cellular remodeling and proliferation [27,28]. The harmful involvement of oxidized proteins in the etiology of Alzheimer's disease has recently been hypothesized [29]. Furthermore, ROS plays a role in diseases like arthritis, diabetes, cancer, and cardiovascular disease [22,23,[30], [31], [32], [33]].

Given the widespread use of proflavine and acriflavine compounds in the treatment of infected wounds, as antimalarial and antiprotozoal agents, and as an anticancer agent, it seemed prudent to conduct toxicity tests on macromolecules, particularly enzymes, in conditions where photoexcitation in the presence of metals could be expected. In this study, we investigated the effect of photo-illuminated proflavine on protein degradation and loss of proteolytic activity of the enzyme trypsin through the generation of hydroxyl radicals, because there is no report in the literature on the deactivation of the enzyme by photo-illuminated proflavine. Additionally, it is important to look at how proflavine affects the structure and function of prospective proteolytic enzymes. The findings of this study might contribute to a better understanding of proflavine's negative effects on protein and enzyme breakdown caused by oxygen-free radicals.

## 2 Materials and methods

### 2.1 Materials

Proflavine (Hemi sulfate salt; CAS-No. 1811-28-5; purity more than 98 %), bovine serum albumin (BSA; EC No.232-936-2; fraction V; purity 98 %), and superoxide dismutase (CAS No.9054-89-1) were purchased from Sigma Chemical Co., Saint Louis, MO, USA. Trypsin crystalline powder (CAS No. 9002-07-7; purity 98 %) and nitro blue tetrazolium (NBT) were purchased from SISCO Research Laboratories, Bombay, India. All other chemicals were of analytical grade. All solutions were prepared fresh. Just before the experiment, proflavine was dissolved in distilled water to make a 1 mM stock solution. All solutions were made in the dark to avoid photo-isomerization of proflavine.

### 2.2 Detection of hydroxyl radicals (^•^OH)

The production of hydroxyl radicals was quantified using a modified method of Halliwell and Gutteridge's [34]. A 0.5 mL reaction mixture comprising 10 mM sodium phosphate buffer pH 7.4, 2 mM deoxyribose, and various concentrations of proflavine was incubated with 800 lx of cool fluorescent light for five hours at 37 °C. The reaction mixture was then heated at 100 °C for 30 min before being cooled to measure absorbance at 532 nm against a blank that did not contain proflavine. To prevent the production of hydroxyl radicals, hydroxyl radical scavengers such as mannitol, thiourea, and sodium formate were utilized as inhibitors. Experiments were carried out three times, with the mean data being recorded each time.

### 2.3 Proflavine induced BSA degradation and trypsin digestion assay

Schroeder method was used for protein degradation and a trypsin enzymatic assay [35]. A total volume of 1 mL of 10 mM sodium phosphate buffer, pH 7.4, with 2 mg/mL of BSA and different doses of proflavine with or without 200 μM CuCl_2_ was used in the reaction mixture. The samples were then treated with 10 μg trypsin for 30 min at 37 °C after being illuminated with fluorescent light. 0.1 mL of 10 mM EDTA and 0.5 mL of 10 % trichloroacetic acid (TCA) were used to stop the reaction. The samples were then centrifuged for 10 min at 2500 rpm to remove the undigested protein (precipitate), and the TCA soluble material in the supernatant was used to determine the acid-soluble peptide using the Lowry et al. [36] method, with the color development being read at 660 nm against a reagent blank. Beckman DU-40 spectrophotometer was used for measuring absorbance.

### 2.4 BSA and trypsin degradation by proflavine and SDS-gel electrophoresis

At different time intervals, the reaction mixture in a total volume of 1 mL 10 mM sodium phosphate buffer pH 7.4 containing either BSA (1 mg/mL) or heat-inactivated trypsin (2 mg/mL) plus proflavine in the presence or absence of 200 μM Cu(II) were incubated with fluorescent light at room temperature. To avoid auto-proteolysis, the trypsin was boiled for 3 min before being used in the procedure. Free radical scavengers such as sodium azide, potassium iodide, sodium benzoate, sodium formate, thiourea, and mannitol were added at a concentration of 50 mM to the protein-proflavine reaction mixture in several tests. The reaction was stopped with 0.025 mL of SDS-sample dye, which consisted of 62.5 mM Tris-HCl pH 6.8, 10 % (v/v) glycerol, 2 % (v/v) β-mercaptoethanol, and 0.001 % (w/v) bromophenol blue. The samples were boiled for five minutes before being loaded with 10 mg of BSA in each well. According to the Laemmli [37] method, the samples were separated on 10 % (w/v) SDS-polyacrylamide gels. In the case of trypsin, 10 μg protein was applied to each lane, and the samples were separated on SDS-polyacrylamide gels containing 15 % (w/v) SDS. After electrophoresis, the gels were stained with silver nitrate staining, as described by Nesterenko et al. [38].

### 2.5 Spectroscopy

Fluorescence spectra were generated using a Shimadzu spectrofluorometer (Tokyo, Japan). 0.7 μM of BSA and lysozyme were stimulated (λ_max_) at 280 nm for tryptophan fluorescence quenching. Fluorescence spectra were read between 300−400 nm with 10 nm of emission slit. Appropriate controls containing native (untreated proteins) were run, and corrections were made wherever necessary. All fluorometric spectral studies were carried out at pH 7.4 and room temperature.

### 2.6 Statistical analysis

The data were examined to utilize SPSS version 22 statistics software. The results are illustrated as the mean ± standard error of the mean (SEM). P values <0.05 were supposed statistically significant. The results shown are mean values of three independent experiments done in triplicate under the same conditions to determine the accuracy of the results and to ensure their reproducibility.

## 3 Results

### 3.1 Generation of hydroxyl radical (^•^OH) by proflavine upon illumination with fluorescent light

**Fig. 1:**
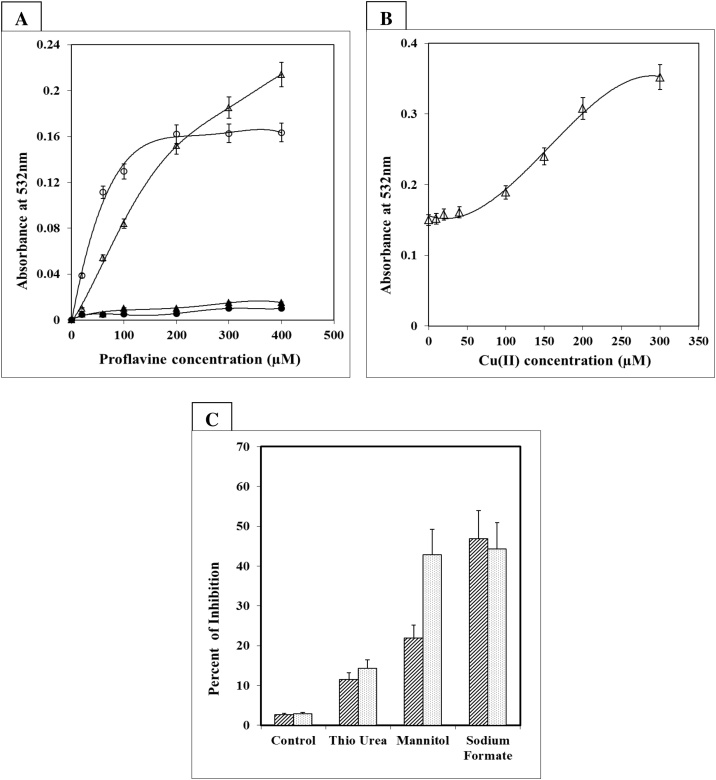
**Generation of hydroxyl radical (.OH) by photoactivation of proflavine.** In a total volume of 0.5 mL of 10 mM sodium phosphate buffer pH 7.4, containing 2 mM deoxyribose incubated with, (A) varying concentration of proflavine (0–400 μM) with 100 μM Cu(II) in light (-△-) and dark (-▲-). (B) 200 μM proflavine with varying concentration of Cu(II) in light (-△-). (C) 50 mM of either thiourea, sodium formate, or mannitol, with 200 μM proflavine () or with 200 μM proflavine and 100 μM Cu(II) (). All reactions were incubated for five hours in the fluorescent light. Each incubation reaction was done in triplicate and the experiments were repeated twice. Means values ± standard deviations are plotted. *P < 0.05 is considered significant.

The possibility of reactive oxygen species like the hydroxyl radical (·OH) being involved in the Fenton reaction was investigated, especially in the presence of metal ions like Cu (II), which is known to engage in the reaction. As shown in Fig. 1A, the formation of hydroxyl radical by light illuminated proflavine with or without Cu (II) was monitored. Increased proflavine and copper concentrations both increased the generation of hydroxyl radicals (Fig. 1B). Various hydroxyl radical scavengers were utilized in the reaction to monitor the specificity of the hydroxyl generation. When deoxyribose degradation was performed in the presence of several hydroxyl radical scavengers such as thiourea, sodium formate, and mannitol, sodium formate showed the greatest inhibition of 46 percent, followed by mannitol, and very little with thiourea (Fig. 1C). When Cu (II) was added to the reaction mixture with proflavine, the inhibition was stronger.

### 3.2 Protein degradation mediated by photo-illuminated proflavine in presence of copper

**Fig. 2:**
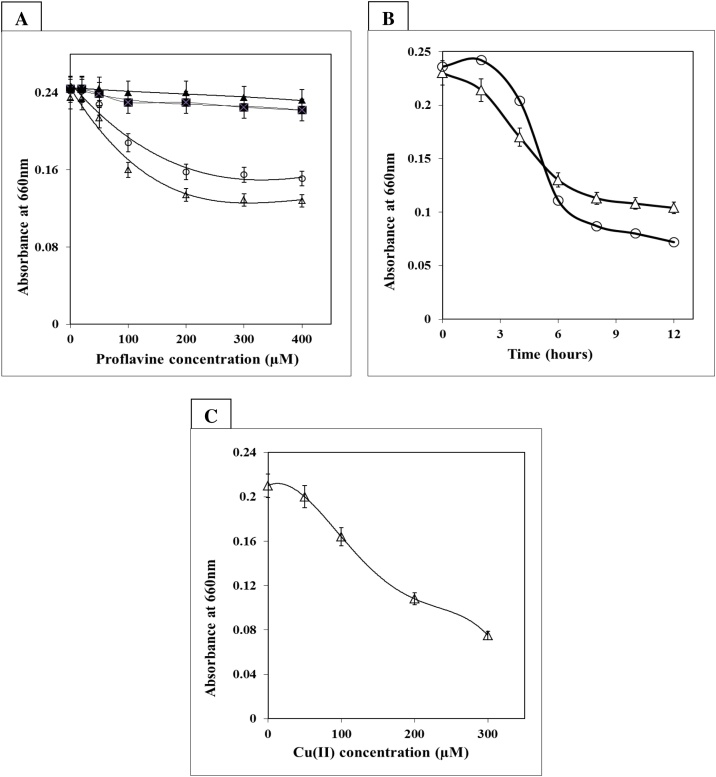
**Protein degradation mediated by photo-illuminated proflavine in presence of copper (II).** BSA (2 mg/mL) was incubated with (A) increasing concentration of proflavine alone in light (-△-) and dark (-▲-), or increasing concentration of proflavine with 100 μM Cu(II) in light (-○-) and dark (-●-). (B) Different time intervals with 200 μM of photoexcited proflavine (-△-) alone, or with 200 μM of photoexcited proflavine and 100 μM Cu(II) (-○-) and (C) 200 μM proflavine in the presence of 0, 50, 100, 200 and 300 μM Cu(II) for six hours (-△-). The TCA soluble product was measured after BSA was treated with 10 μg of trypsin for 30 min at 37 °C.

BSA induced a steady decrease in the synthesis of acid-soluble material after irradiation with proflavine or proflavine plus Cu (II) in fluorescent light, as measured by trypsin digestion assay. However, in the absence of light, no such effect was observed (Fig. 2A). Furthermore, in the reaction buffer, a decrease in the tryptic hydrolysis of the proflavine-treated protein was seen in a concentration and time-dependent manner (Fig. 2A, B). Furthermore, increasing Cu (II) concentration appears to improve protein modification, as seen by a decrease in protein breakdown evaluated spectrophotometrically by trypsin assay (Fig. 2C).

### 3.3 Proflavine, with or without Cu(II), alters protein structure

**Fig. 3:**
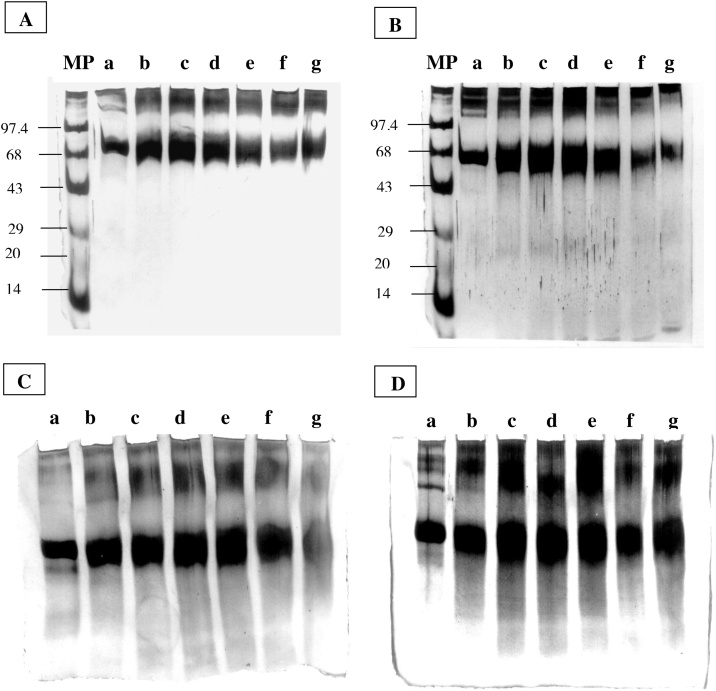
**SDS-PAGE analysis of proflavine induced protein structure.** BSA (2 mg/mL) incubated with proflavine with or without copper at different time intervals in fluorescent light at room temperature. 10 μg of protein was applied in each lane of 10 % SDS- PAGE and analyzed. (A) Increasing concentration of proflavine (10−300 μM), Lane b–g; BSA alone (Lane a). (B) Increasing concentration of proflavine (10−300 μM) with 200 μM Cu (II); Lane b–g; BSA alone (Lane a). (C) Time course (2−12 h) of 200 μM proflavine; (Lane b–g); BSA alone (lane a). (D) Time course (2−12 h) of 200 μM proflavine in presence of 200 μM Cu(II); (Lane b–g). (Lane a) BSA alone. The gels were stained with silver nitrate. (A) and (B) the reaction mixtures were incubated for six hours. Reaction mixtures were incubated in fluorescent light at room temperature before electrophoresis.

BSA was incubated with increasing doses of proflavine and subjected to light, as shown in Fig. 3A. BSA's SDS-PAGE profile revealed considerable protein alteration and degradation. After five hours of exposure to fluorescent light, cleavage and/or modification of a protein by proflavine resulted in smearing and degradation as the sample near the top of the gels was reduced, followed by a broadening of the band at monomer position due to fragmentation to slightly smaller peptides. Furthermore, the presence of Cu(II) accelerated the breakdown of proteins into smaller fragments (Fig. 3B). In addition, the capacity of increasing photo illumination time with proflavine in the presence or absence of Cu(II) to degrade BSA was examined (Fig. 3C and D). When the light exposure period to proflavine was doubled, there was a significant increase in protein breakdown. Increased light exposure duration to proflavine in the presence of Cu(II) appears to degrade the protein more robustly than in the absence of Cu(II). BSA was not degraded in the absence of light or Cu (II) alone (data not shown).

### 3.4 Photo-illuminated proflavine disrupts enzyme activity

**Fig. 4:**
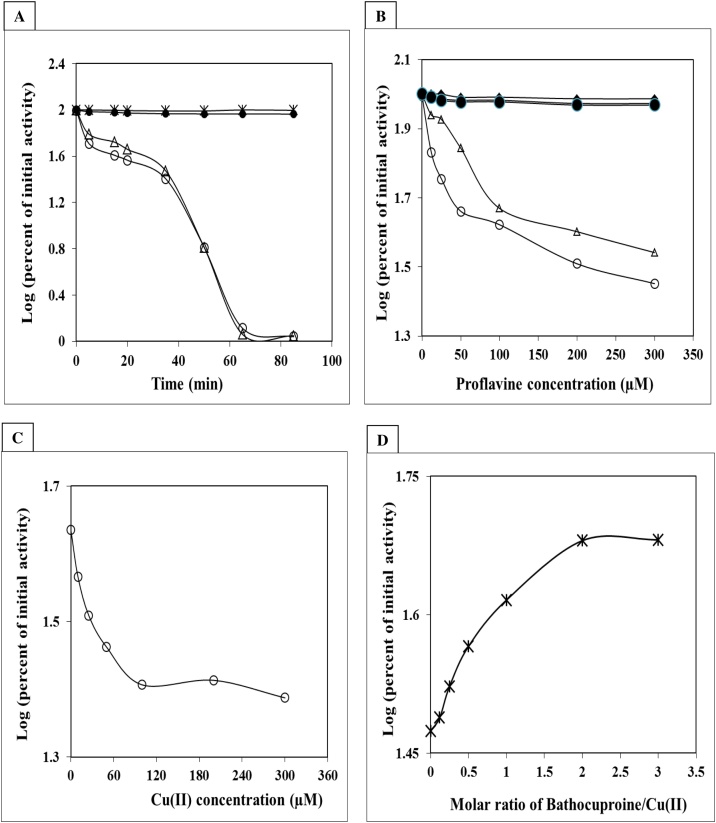
**Photo-illuminated proflavine disrupts enzyme activity.** The inactivation of the proteolytic activity of trypsin was analyzed by photo-illuminated proflavine with or without Cu(II). (A) Trypsin was incubated with 200 μM proflavine (-△-) and 200 μM proflavine with 100 μM Cu(II) (-○-); Also trypsin alone (-X-) and trypsin with 100 μM Cu (II) were exposed to fluorescent light before its incubation with BSA. (B) Increasing concentration of proflavine in light (-△-) and dark (-▲-) and increasing concentration of proflavine with 100 μM Cu(II) in light (-○-)and dark (-●-). (C) 200 μM proflavine and increasing concentration of Cu(II). (D) 200 μM proflavine plus 100 μM Cu(II) and increasing concentration of bathocuproine. Reaction volume of 2 mL contained 15 μg of trypsin in 10 mM sodium phosphate buffer pH 7.4 at 25 °C. In (B), (C), and (D), reactions were incubated for 35 min under fluorescent light. Enzyme activity was assayed as described in the method section.

We investigated whether photo-illuminated proflavine with or without Cu (II) disrupted the enzymatic activity of trypsin, a well-known proteolytic enzyme. The release of acid-soluble peptides from the protein is examined to see if the enzyme is inactivated. Before incubation with BSA (as a substrate), trypsin was treated with proflavine and exposed to fluorescent light (Fig. 4A). The activity of trypsin was lowered by about 40 % after 35 min of exposure to light and proflavine. Proflavine in the reaction mixture was likewise found to suppress trypsin activity in a concentration-dependent manner (Fig. 4B). Cu (II) was added to the photo-illuminated process and caused a quick decrease in enzyme activity after 35 min; however, increasing the Cu (II) concentration did not impact the activity. This could be attributed to an increase in the molar ratio of Cu (II) to proflavine (Fig. 4C). When bathocuproine was introduced to the Cu (II)-containing process, the enzyme inactivation was greatly reduced (Fig. 4D), showing that Cu (I) is involved in the photo-illuminated damage to trypsin.

### 3.5 Degradation of trypsin protein and electrophoretic Analysis

**Fig. 5:**
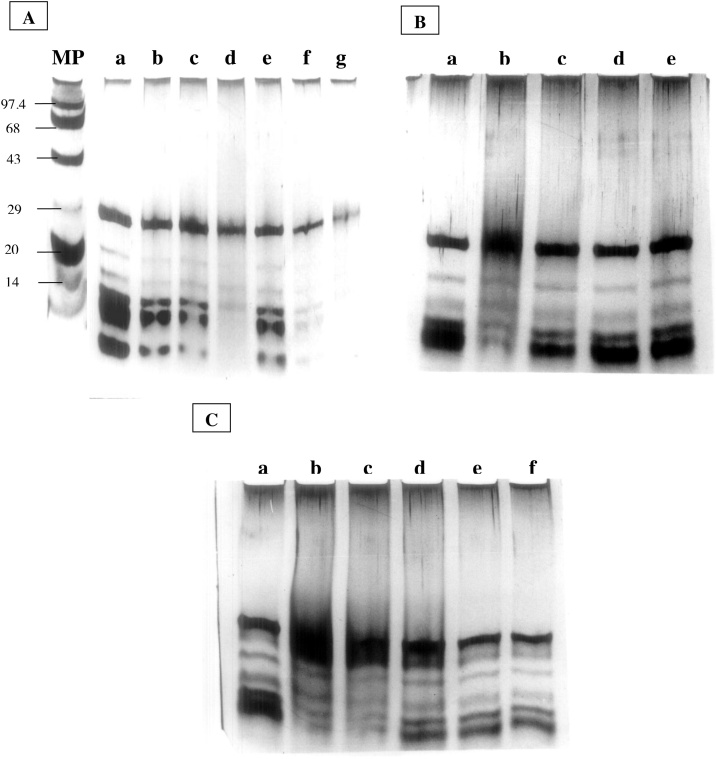
**Degradation of trypsin protein and electrophoretic Analysis.** (A) SDS-PAGE of trypsin incubated for 4, 8, and 12 h with 200 μM proflavine (lane **b-d**) and trypsin incubated with 200 μM proflavine plus100 μM Cu(II) (lane **e-g**). Lane **a** contains trypsin incubated alone as a control. (B) Trypsin incubated with 200 μM proflavine and 50 mM of sodium azide, potassium iodide, or thiourea (lanes **c-e**); trypsin with 200 μM proflavine (lane **b**) and trypsin alone (lane **a**). (C) Trypsin alone as a control (lane **a**), trypsin incubated with 200 μM proflavine (lane **b**), trypsin with 200 μM proflavine plus 200 μM Cu(II) (lane **c**), trypsin incubated with 200 μM proflavine and 200 μM Cu(II) plus 50 mM of sodium azide or potassium iodide or thiourea respectively (lanes **d-f**). 10 μg of trypsin was applied in each lane after the treatments and 15 % SDS-PAGE was used for the analysis: In both (B) and (C) the reactions were incubated for eight hours in fluorescent light at room temperature and gels were silver stained after electrophoresis.

In a time-dependent way, photosensitized proflavine appears to cause fragmentation and degradation of trypsin protein. The band intensity of the main 24 kDa subunit of trypsin appears to be decreasing as the incubation period with proflavine increases (Fig. 5A). When the enzyme was treated with proflavine-Cu (II), it degraded faster than when it was incubated without Cu (II). As the incubation period was prolonged, the degradation of the enzyme increased as well, as seen by a decrease in the protein's band intensity. Following that, we looked at the role of free radicals like (^1^O_2_, ^3^O_2_, and ^•^OH) in the degradation of trypsin protein. We did this by incubating the trypsin plus proflavine reaction mixture with free radical scavengers including sodium azide, potassium iodide, and thiourea. Surprisingly, all of these scavengers appear to protect the enzyme from destruction (Fig. 5B). Additionally, the scavenging effects of free radicals were investigated when trypsin was treated with proflavine in the presence of Cu (II). Proflavine and/or Cu(II) increased trypsin degradation, but when those hydroxyl radical scavengers were added, the degradation was reduced, as the band intensities were found to be similar to controls (Fig. 5C).

### 3.6 Fluorescent quenching studies on protein using proflavine

**Fig. 6:**
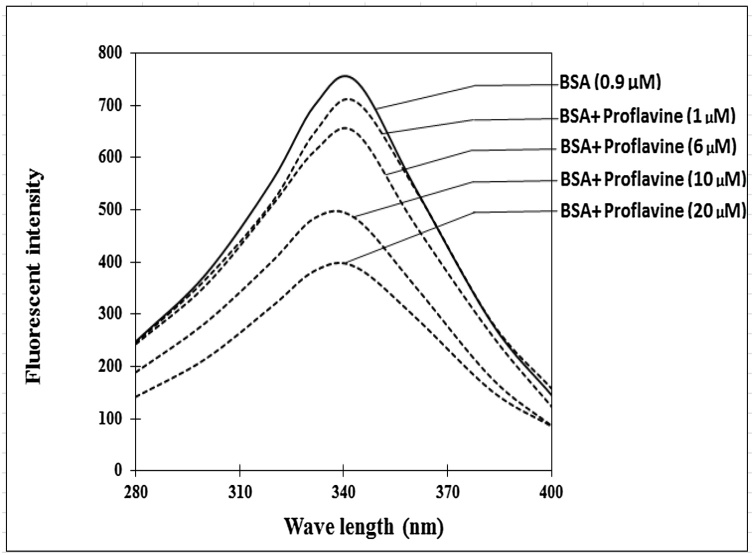
**Fluorescent emission spectra of 0.9 μM BSA incubated with proflavine**. The 2.0 mL reaction mixture containing BSA was incubated with 1–20 μM of proflavine and excited at 280 nm. The fluorescent emission spectra were recorded with an emission slit of 10 nm.

**Fig. 7:**
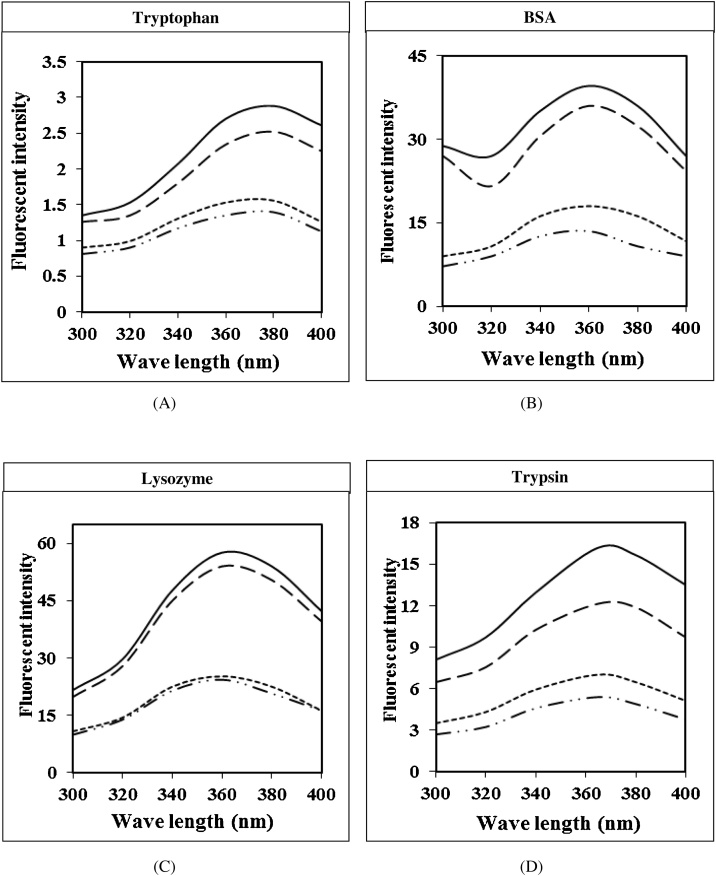
**Fluorescence spectra of tryptophan, BSA, and enzymes complex with proflavine and/or Cu(II).** Reaction mixture contained 2 mL, 10 mM sodium phosphate buffer, pH 7.4, containing 0.7 μM protein/tryptophan (), protein/tryptophan with Cu(II) (12.5 μM) (**– – –**), protein/tryptophan with proflavine (25 μM) (·······), and protein/tryptophan with proflavine and Cu(II) (). The excitation wavelength was 280 nm and the emission slit was 10 nm. (A) Tryptophan alone (B) BSA: the blue shift from 380 nm to 356 nm with a weak peak. (C) Lysozyme: the blue shift from 380 nm to 358 nm with a broad peak. (D) Trypsin: the blue shift from 380 nm to 373 nm with a broad peak. All were compared to free tryptophan.

With proflavine concentrations ranging from 0 to 20 μM, the fluorescence quenching of BSA was measured (Fig. 6). As the quantity of proflavine grew, so did the amount of fluorescence quenching. When 20 μM proflavine was incubated with BSA, about 50 % of the fluorescence was quenched. Because we predicted proflavine to attach to BSA near or on tryptophan residues, we examined the binding spectra of proflavine to BSA and tryptophan alone (Fig. 7A, B). We also studied fluorescence quenching in lysozyme and trypsin, two additional proteins with different tryptophan contents (Fig. 7C). When compared to the fluorescence of the free amino acid, all of these proteins demonstrated a change in the maximum wavelength for tryptophan fluorescence to a lower wavelength. The fluorescence quenching for BSA and lysozyme was 49 % and 56 %, respectively, demonstrating that the quenching increased as the protein's tryptophan residues increased. The quenching was shown to increase with increasing proflavine concentration in all cases. The effect of proflavine with or without Cu(II) on the fluorescence spectra of trypsin was also investigated. In the case of unbound amino acids, there was a shift in the maximum fluorescence wavelength to a lower wavelength (Fig. 7D). Free tryptophan quenches fluorescence by 63 percent when combined with proflavine or proflavine plus Cu(II) (Fig. 7A). This demonstrates that BSA is one of the restricted proteins to which proflavine can bind that influences tryptophan residue fluorescence.

## 4 Discussion

Proflavine has been linked to several potentially harmful side effects, including mutagenicity, apart from its widespread usage as an antiseptic and disinfectant. Proflavine and its acridine derivatives are shown to infiltrate cells and attach to protein/enzyme and DNA, causing physiological dysfunction and cell death [39,40]. When exposed to visible light, proflavine releases a variety of reactive oxygen species, which cause double-stranded breaks in DNA [6,14].

Previously, we observed that light-induced proflavine generated superoxide ion (O_2_^•^^−^), and that caused structural changes in proteins which were amplified when the divalent metal ion Cu (II) was added [15]. In this paper, we describe hydroxyl radical as a major source generated by light-activated proflavine in presence of Cu (II), which has detrimental effects on enzyme activity and structural alterations in the protein. A robust increase in the synthesis of hydroxyl radical (^•^OH) corresponding to an increasing concentration of photo-activated proflavine and Cu(II) was observed, even in the absence of any macromolecules. This is in contrast to the majority of prior investigations, which have indicated that free radical generation from light-induced proflavine is only achievable in the presence of macromolecules like DNA [4]. The specificity of the formation of ^•^OH, radicals in the reaction are validated using hydroxyl radical specific inhibitors.

Copper was employed in our research since it is an essential trace element that is found throughout the body. It's present in a range of tissues, including the liver and kidney, where it's abundant [16]. In the field of biochemistry, metal particles are employed for drug development, diagnostics, delivery, bioimaging, and treatments [41]. Many metalloproteins, including ceruloplasmin, Cu, Zn superoxide dismutase, cytochrome C oxidase, tyrosinase, and ascorbate oxidase, have copper as the redox-active core. Metal ions, such as Cu(II), are known to engage in Fenton-like reactions that stimulate the generation of ROS. During photo-illumination of proflavine, the presence of Cu (II) increased the formation of hydroxyl radicals. Proteins are the primary targets of reactive oxygen species, which can lead to oxidation and influence cellular remodeling and growth [42]. Under aerobic conditions, BSA got fragmented when exposed to ^•^OH and O_2_^•^^−^ [43]. We investigated the effect of reactive oxygen species produced by photo-illuminating proflavine on BSA. The protein was significantly degraded by proflavine when exposed to light irradiation. The inclusion of Cu(II) in the reaction accelerated the rate of degradation substantially. As our results indicate, photo-illuminated proflavine generated significant structural and functional changes in the protein.

Previous studies have shown that in vivo and in vitro interactions of proteins with oxygen free radicals resulted in increased hydrophobicity due to amino acid modifications, potentially increasing proteolysis susceptibility [[43], [44], [45], [46]]. Furthermore, Wolff and Dean [44] demonstrated that ·OH attacked proteins which caused conformational changes in the proteins, increasing their vulnerability to enzymatic proteolysis. However, interestingly, in this investigation, BSA susceptibility to tryptic proteolysis was reduced in the presence of proflavine and Cu(II), indicating the possibility of alteration of certain amino acids, such as lysine and arginine, which are trypsin-recognized amino acid residues. Others have observed that certain amino acids are modified after being exposed to radicals [47], confirming our findings. We previously postulated two putative ROS formation pathways from photoexcited proflavine: triplet (**^3^**O_2_) and singlet (**^1^**O_2_) radicals, and peroxide radicals (O_2_^•^^−^), which may produce (·OH) radicals. All of these oxygen and hydroxyl radicals are thought to play a potential role in protein breakdown [15].

Many enzymes accumulate catalytically inactive or less active, more heat-labile forms, which is an indicator of cellular aging [48,49]. Since proflavine inhibits substrate binding by binding competitively to trypsin [50]. Therefore, we used photo-activated proflavine in the presence of Cu (II) to inactivate the monomeric non-glycoprotein enzyme, trypsin. The rate of formation of the enzyme-inhibitor (proflavine) complex is equivalent to that of the enzyme-substrate complex, thus a high concentration of substrate is required for the production of the enzyme-substrate complex when an inhibitor such as proflavine is present. For this reason, BSA, the trypsin substrate in our investigation, was always utilized in excess. Despite this, trypsin's proteolytic activity was shown to be significantly reduced, most likely due to free radical-mediated damage to specific amino acids in the protein caused by proflavine. When Cu (II) was added to the process, the loss of trypsin activity was even more significant.

The steady decrease in emission intensity demonstrated that proflavine either directly binds to tryptophan or alters the conformation of BSA, resulting in tryptophan shielding, as revealed by the emission spectra of BSA recorded after the addition of increasing proflavine concentrations. Changes in the aromatic side-chain composition, such as a loss of conjugation from tryptophan or other aromatic amino acids, are reflected in the decreased fluorescence [51]. For fluorescence investigations, BSA and other proteins with different levels of tryptophan, such as lysozyme and trypsin, were employed. In all cases, a change in λ_max_ for tryptophan fluorescence was detected, along with quenching, indicating that proflavine binds to these proteins around tryptophan residues. The shift in λ_max_ was maximum with BSA followed by lysozyme and minimum with trypsin. This indicates that trypsin's tryptophan is in the least hydrophobic environment possible.

Our study is significant since a wide range of proflavine and acriflavine compounds have been produced and used clinically as antibacterial, antimalarial, and antiprotozoal medicines, [4,11,52]. In addition, work on developing novel proflavine compounds and derivatives have begun, which could have interesting anticancer properties [53].

Taken together, photoinduced-proflavine seems to generate an excess of hydroxyl radicals that resulted in protein degradation and loss of enzyme activity. Photoexcitation of proflavine in the presence of metals ions especially Cu(II) augmented the generation of hydroxyl radicals. Proflavine resulted in oxidative modifications of protein and enzyme structure and function. The findings of this study might contribute to a better understanding of proflavine's harmful effects on protein and enzyme breakdown caused by oxygen-free radicals. However, further research into proflavine's harmful role in oxidizing and destroying proteins and enzymes is needed, as this substance may increase the chance of acquiring a variety of disorders.

## Author contribution

Work design and conceptualization were done by MKG and MSO. All the work was carried out by MKG and MSO. Data analysis was done by HO and AM. The manuscript was prepared and written by MSO and ASA.

## Conflict of interest

The authors declare no conflict of interest.

## Declaration of Competing Interest

The authors report no declarations of interest.
